# Study on the influence of voids on high-rise building on the wind environment

**DOI:** 10.1007/s12273-019-0584-7

**Published:** 2019-12-05

**Authors:** Yangluxi Li, Lei Chen

**Affiliations:** Welsh School of Architecture, Cardiff University, Cardiff, UK

**Keywords:** high-rise buildings, opening, void, concavity, CFD simulation, building efficiency, wind environment

## Abstract

The purpose of this study was to investigate the effects of voids in tall buildings on the surrounding wind environment. With the development of modular technology, there has been a new method of building high-rise buildings. Currently, more and more high-rise buildings often use void spaces to reduce the wind resistance and utilize wind turbines by using wind power to create sky gardens. In this study, CFD (computer fluid dynamic) technology was used to simulate the wind environment around the buildings. The research focuses on the size, distribution and quantity of the concavity, which usually is defined as sky gardens. It is found that when the area of the opening is the same, the more number of opening, the more strengthened and distributed vertical wind velocity behind the building can be. The wind shadow area at the pedestrian height is further reduced. For holes distribution, the optimum ratio of the spacing between concavities to the void size for wind environment of tall buildings ranges from 1 to 3, which can disperse the surrounding heat in more efficiency and weaken the wind velocity in the lowest level. Therefore, in high-rise buildings, the number and distribution of the openings will have different effects on the wind environment around the buildings.

### Keywords high-rise buildings,
opening,
void,
concavity,
CFD simulation,
building efficiency,
wind environment

### 1 Introduction

Pedestrian wind security and comfort are crucial requirements for a city (Moonen et al. 2012). Taking consideration of its importance, many governmental institutions released new building policy in according with airflow safety and comfort evaluation researches, which could improve surrounding comfort and safety (Du et al. 2012, 2016).

Wind damage in cities was usually caused by tall buildings because they often produced unpredicted or dangerous wind environment at the pedestrian level (Melbourne and Joubert 1971; Murakami et al. 1986). In spite of uncomfortable environment for pedestrian was usually attributed to the high wind velocity, wind-related environmental problems have transmitted from dangerous high wind velocity to disadvantageous low wind speeds (Chetwittayachan et al. 2002; Goyal and Sidhartha 2002; Tsang et al. 2012). For example, Hong Kong's dense high-rise buildings and large platform structures had greatly reduced wind penetration, resulting in poor low wind speeds at the pedestrian level (Chetwittayachan et al. 2009; Goyal and Sidhartha 2012). Extensive stagnant air promoted airborne pathogens, such as the SARS virus (Severe Acute Respiratory Syndrome), posing a threat to public health (Yu et al. 2004). In addition, the average wind speed was lower than the minimum average wind speed of 1.5 m/s required by Hong Kong's air circulation assessment, which will cause outdoor heat discomfort to pedestrians, especially during the hot summer months (Ng 2009)

Therefore, people have increasingly focused on low airflow velocity districts around high-rise buildings, which may result in poor outdoor air quality. Research done so far with respect to airflow condition around the construction has been performed for more than 50 years. Blocken and Carmelie (2004) reviewed a lot of researches in terms of outdoor human comfort degree studies since from the 1960s and found studies about airflow environment at pedestrian level mainly concentrated on the poor surroundings caused by strong wind flow around high-rise buildings (Wiren 1975; Stathopoulos and Storms 1986; Uematsu et al. 1992; Jamieson et al. 1992;

**List of symbols**

| | | | |
|---|---|---|---|
| $C_\mu$, $C_{1\varepsilon}$, $C_{2\varepsilon}$, $\sigma_k$, $\sigma_\varepsilon$ | constants in the standard $k$–$\varepsilon$ model | $\bar{u}$ | averaged fluid velocity |
| $I$ | turbulence intensity | $u^*_{\mathrm{ABL}}$ | ABL friction velocity |
| $k$ | turbulence kinetic energy | $U_\mathrm{r}$ | reference wind velocity |
| $l$ | turbulence length scale | $\tilde{\nu}$ | modified turbulent viscosity |
| $L$ | associated size | $x$, $y$, $z$ | Cartesian co-ordinates |
| $P$ | mean static pressure | $z_0$ | roughness length(or height) |
| $Re_{D_\mathrm{H}}$ | Reynolds number | $z_\mathrm{G}$ | gradient height |
| $S_i$ | source item | $\delta_{ij}$ | Kronecker=1, delta for $i = j$, and =0 for $i \neq j$ |
| $u_i$ | mean wind velocity at $i$ direction | $\varepsilon$ | dissipation rate of $k$ |
| $u_j$ | mean wind velocity at $j$ direction | $\kappa$ | Von Kaman constant |
| $u_\mathrm{G}$ | mean wind velocity at the gradient height | $\mu_\mathrm{t}$ | turbulence viscosity |
| $u'$ | turbulent fluctuating velocity | $\rho$ | fluid density |
| $u'_i$ | fluctuating velocity component at $i$ direction | | |

Stathopoulos and Wu 1995; To and Lam 1995; Kubota et al. 2008). Many impact factors such as shape, space layout, orientation of buildings and street canyons were studied, and the results provide precious insights in view of the pedestrian-level wind environments around buildings (Stathopoulos et al. 1992; Visser et al. 2000).

Nevertheless, most of these studies only concentrated on small regions with high wind speed such as building corner nearby. For a group of buildings or row of constructions, some researches had been done primarily investigated the passage between constructions. The height and width of the building, the separation between the building and the podium ventilation effects were performed to be studied by the different architectural shapes consisting of single building, two buildings, a row of building and so on. Natural ventilation and pedestrian comfort were also assessed via experimental design and analysis (Chan et al. 2001, 2003). Note that, beyond that there were a few studies to investigate the influences of modern building structures (such as buildings with platforms) on the relatively large area of low flow velocity at the leeward of the building.

Blocken and Stathopoulos (2013) performed a comprehensive investigation in regard to CFD simulation on pedestrian-level wind condition utilizing a normalized mean wind speed ($U/U_\mathrm{r}$) rather than speed ratio $K$ because it could easily combine specific wind climates to determine wind speed statistics based on the magnitude and frequency of occurrence. This contributed to determine effectively the boundaries of low wind velocity region. For instance, the normalized mean wind speed ($U/U_\mathrm{r}$) about 0.3 was needed so as to achieve a threshold wind velocity approximately 1.5 m/s that was the lowest wind velocity observable at the pedestrian level (Lawson and Penwarden 1975). Therefore, $U/U_\mathrm{r}$ lower than 0.3 was considered as the low wind velocity area which had negative influence on the ventilation. This pedestrian horizontal wind speed of 1.5 m/s threshold was consistent with Ng (2009) as the Hong Kong air circulation assessment criteria, taking into account the environment temperature and solar irradiance at that time. It can be seen from that definition of area utilizing wind climate in Hong Kong was a good method. The normalized mean wind velocity ($U/U_\mathrm{r}$) distribution given here was known as a general result of the characteristics of airflow around the object building.

Recently, a number of international initiative researches have been done focusing on establishing common optimum practice guidelines (e.g. Franke et al. 2007; Tominaga et al. 2008a; Casey and Wintergerste 2000; Blocken and Gualtieri 2012). Several CFD studies have been performed on CFD simulations about pedestrian-level wind situations in constructions and urban (Stathopoulos 2006; Moonen et al. 2012; Blocken et al. 2007; Blocken et al. 2012). Most of them focused on implementing the 3D steady Reynolds-averaged Navier–Stokes (RANS) methodology to perform simulation. In the past, some relevant researches compared the CFD results with the tunnel wind experiments under the same building or urban form to verify its validation (Richards et al. 2002; Stathopoulos 2006; Mochida and Lun 2008; Gadilhe et al. 1993; Stathopoulos and Baskaran 1996; Ferreira 2002; Westbury et al. 2002; Blocken et al. 2008).

This paper investigates the effect of the "voids" integrated on high-rise building on wind characteristic using CFD simulation method under ABL (atmospheric boundary layer) inlet condition. High-resolution mesh coupled steady RANS method are implemented to perform simulations for 14 cases with different holes positions and numbers. The perform indicator is constructed in the basis of the normalized wind velocity of WVA (wind velocity amplification factor) and pollutant concentration CR (concentration ratio). Whole CFD simulation is validated by a wind tunnel measurement by Tsang et al. (2012) of normalized wind speed. This research results could contribute architects to construct sky courts more reasonable and utilize special positions of voids to harvest wind energy to produce power. This paper includes six parts: Section 1 introduces some relevant researches done so far. Sections 2 and 3 describe simulated methodology and wind tunnel measurement validation. Section 4 mainly investigates the simulation results. Section 5 discusses about related future research direction. Section 6 presents final critical conclusions.

### 2 Methodology

#### 2.1 Case settings

The purpose of this study is to explore the number of voids in the high-rise and the impact of the hole distribution on the wind environment around the building. The model is divided into three types of cases for study as shown in Fig. 1.

Case A: This case researches the influence on the wind environment by the numbers of voids in building. Buildings integrated with different holes numbers are considered (5 cases). Under the same area of the whole openings, the voids number ranges from 3 to 9 and the corresponding heights are $L/3$, $L/5$, $L/7$, $L/9$, individually. In each case, the building height and holes area remain identical to each other.

Case B: Buildings with different "holes" distribution condition. The area of concavity is divided equally into three groups, and the ratio $R$ is the ratio of the gap between two voids to the height of hollows ranges from 1 to 4 shown in Fig. 1. Apart from this, the number of voids remains unchanged in any cases, furthermore, the holes move from center to the both end of the sides of building in different cases B.

Case C: In this case, the openings mainly concentrate upper or lower part of building which differs from case B that hollows distributed at the middle of building. Aspect ratio changes from 0 to 2 in corresponding upper holes cases which are represented as case CU0 to case CU2. It should be noted that case C just changed the openings distribution maintaining identical quantity and area of voids with case B. Table 1 shows the features for different cases.

The method of counting statistics data is shown in Fig. 1. It can be clearly seen that in order to measure the vertical ventilation condition behind building, a vertical line is created to record the wind velocity where distances the building 10 m along the centre line. In addition, so as to assess pedestrian level wind condition, a horizon line is performed at the height of 1.5 m walking level along the central axis leeside of the building to monitor wind velocity.

Perform indicators:

Normalized wind velocity of wind velocity amplification factor (WVA) is defined as follows:

$$\mathrm{WVA} = \frac{U\,(\text{mean wind speed})}{U_{\mathrm{ref}}\,(\text{mean inlet wind speed})}$$

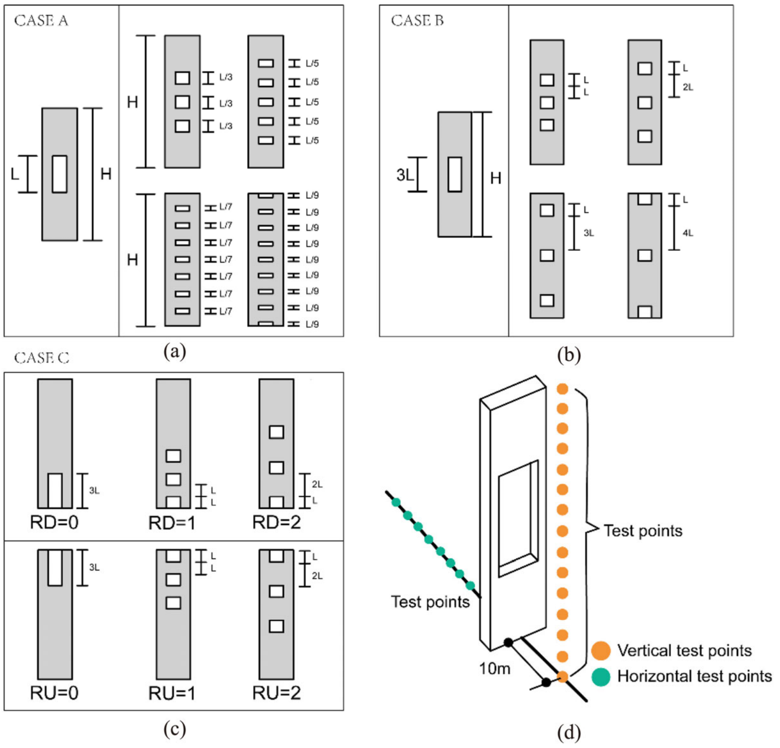

**Fig. 1** Research cases description. (a) Case A: different numbers of holes; (b) case B: distribution of holes; (c) case C: distribution of holes effect; (d) test points line location drawing

**Table 1** Case settings

| Name | Sub-name | Feature | Schematic diagram |
|---|---|---|---|
| | Case A0 | | |
| | Case A3 | | |
| Case A | Case A5 | 1. Under the same area of the whole openings, the voids number ranges from 3 to 9 and the corresponding heights are *L*/3, *L*/5, *L*/7, *L*/9<br>2. Nomenclature: case A+number of holes |  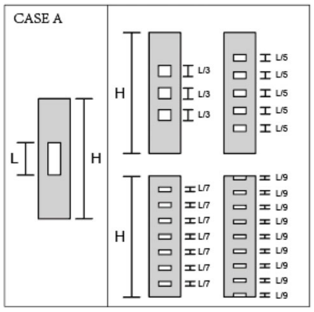 |
| | Case A7 | | |
| | Case A9 | | |
| | Case B0 | | |
| | Case B1 | | |
| Case B | Case B2 | 1. The area of cavity is divided equally into three groups and the ratio *R* is the ratio of the gap between two voids to the height of holes ranges from 1 to 4<br>2. Nomenclature: case B+value of *R* |  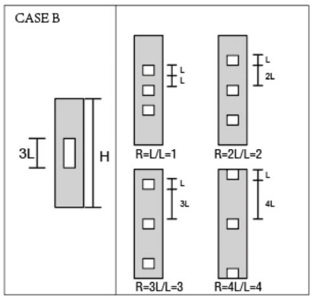 |
| | Case B3 | | |
| | Case B4 | | |
| | Case CU1 | | |
| | Case CU2 | | |
| Case C | Case CU3 | 1. The openings mainly concentrate upper or lower part of building which differs from case B that holes distributed at the middle of building<br>2. Nomenclature: case C+opening position+value of *R* |  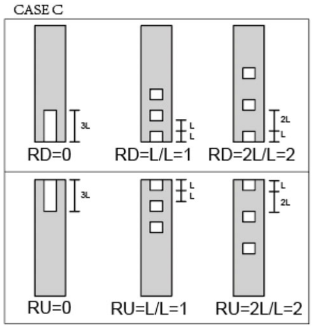 |
| | Case CD1 | | |
| | Case CD2 | | |
| | Case CD3 | | |

Pollutant concentration ratio (CR) is defined as follows:

$$\mathrm{CR}=\frac{\text{pollutant concentration of inlet wind (approch airflow)}}{\text{pollutant concentration along the vertical measurement line}}$$

### 2.2 Governing equation

Entire wind flow field is determined by three governing equations for continuity, momentum and energy. A common method to investigate the wind field is to decompose the governing equations based on the averaged time. Hence, the time-averaged mean flow continuity equation is shown as follows:

$$\frac{\partial(\rho u_i)}{\partial t}+\frac{\partial(\rho u_i u_j)}{\partial x_j}=-\frac{\partial P}{\partial x_i}+\frac{\partial}{\partial x_j}\left(\mu\frac{\partial u_i}{\partial x_i}-\rho\overline{u_i'u_j'}\right)+S_i \quad (1)$$

where $u$ is the airflow velocity and $u_i$, $u_j$ are its components in different directions. $P$ is the mean static pressure, $\rho$ is the air density. $S_i$ is corresponded source item. In Eq. (1), the $\rho\overline{u_i'u_j'}$ is the key to solve equation and considered as Reynolds stress. Moreover, a new turbulence model equation must be constructed to investigate the new item of Reynolds stress.

Turbulent viscosity should be employed to obtain Reynolds stress term. The turbulent stress is expressed as a function of turbulent viscosity. Hence, the key of the whole calculation is to determine the turbulent viscosity. The turbulent viscosity is put forward by Boussinesq's eddy viscosity hypothesis, which establishes the relationship between Reynolds stress and mean velocity gradient as follows:

$$-\rho\overline{u_i'u_j'}=\mu_t\left(\frac{\partial u_i}{\partial x_j}+\frac{\partial u_j}{\partial x_i}\right)-\frac{2}{3}\left(\rho k+\mu_t\frac{\partial u_i}{\partial x_j}\right)\delta_{ij} \quad (2)$$

where $\mu_t$ is the turbulent viscosity, $u_i$, $u_j$ are the average air velocity and $\delta_{ij}$ is Kronecker delta. When $i=j$, $\delta_{ij}=1$; when $i\neq j$, $\delta_{ij}=0$. $k$ is the turbulent kinetic energy:

$$k=\frac{\overline{u_i'u_j'}}{2}=\frac{1}{2}\left(\overline{u'^2}+\overline{v'^2}+\overline{w'^2}\right) \quad (3)$$

It can be seen from the above that the key to achieve turbulent flow is how to determine $\mu_t$ after implementing Boussinesq's hypothesis. Currently, the two-equation model is most widely used in engineering. The basic two-equation model is the standard $k$–$\varepsilon$ model in which the equations of turbulent kinetic energy $k$ and dissipation rate $\varepsilon$ are used respectively.

Standard $k$–$\varepsilon$ model refers to an equation combining turbulent kinetic energy $k$ with dissipation rate $\varepsilon$ which is defined in the basis of the equation of turbulent kinetic energy $k$. This model was first proposed by Launder and Spalding (1974). In the model, the turbulent dissipation rate is estimated as

$$\varepsilon = \frac{\mu}{\rho}\overline{\left(\frac{\partial u_i'}{\partial x_k}\right)\left(\frac{\partial u_i'}{\partial x_k}\right)} \tag{4}$$

Furthermore, turbulent viscosity $\mu_t$ can be expressed as a function of $k$ and $\varepsilon$:

$$\mu_t = \rho C_\mu \frac{k^2}{\varepsilon} \tag{5}$$

where $C_\mu$ is an empirical constant. In the standard $k$–$\varepsilon$ model, $k$ and $\varepsilon$, are solved by the following transport equation:

$$\frac{\partial(\rho k)}{\partial t} + \frac{\partial(\rho k u_i)}{\partial x_i} = \frac{\partial}{\partial x_j}\left[\left(\mu + \frac{\mu_i}{\sigma_k}\right)\frac{\partial k}{\partial x_j}\right] + G_k + G_b - \rho\varepsilon - Y_M + S_k \tag{6}$$

$$\frac{\partial(\rho \varepsilon)}{\partial t} + \frac{\partial(\rho \varepsilon u_i)}{\partial x_i} = \frac{\partial}{\partial x_j}\left[\left(\mu + \frac{\mu_i}{\sigma_\varepsilon}\right)\frac{\partial \varepsilon}{\partial x_j}\right] + C_{1\varepsilon}\frac{\varepsilon}{k}(G_k + C_{3\varepsilon}G_b) - C_{2\varepsilon}\rho\frac{\varepsilon^2}{k} + S_\varepsilon \tag{7}$$

Among them, $G_k$ is the term of turbulent kinetic energy $k$ caused by mean velocity gradient, $G_b$ is the term of turbulent kinetic energy $k$ caused by buoyancy, $Y_M$ represents the fluctuation expansion in compressible turbulence. $C_{1\varepsilon}$, $C_{2\varepsilon}$, and $C_{3\varepsilon}$ are empirical constants. $\sigma_k$ and $\sigma_\varepsilon$ are the Prandtl numbers corresponding to turbulent kinetic energy $k$ and dissipation rate $\varepsilon$, respectively. $S_k$ and $S_\varepsilon$ are user-defined source items. In accordance with the recommended values of Launder and Spalding (1974) the model constants can be obtained as follows:

$$C_{1\varepsilon} = 1.44,\ C_{2\varepsilon} = 1.92,\ C_\mu = 0.09,\ \sigma_k = 1.0,\ \sigma_\varepsilon = 1.3 \tag{8}$$

According to above standard viscosity model, RNG (renormalization group) $k$–$\varepsilon$ model is proposed by Yakhot and Orzag. In RNG $k$–$\varepsilon$ model, small-scale eddy motions are systematically replaced by large-scale eddy in the governing equation. The $k$ and $\varepsilon$ equation can be obtained as follows which are similar with the standard $k$–$\varepsilon$. In this study, RNG model is chosen to perform the simulation which has been proved to be an efficient model for similar research.

$$\frac{\partial(\rho k)}{\partial t} + \frac{\partial(\rho k u_i)}{\partial x_i} = \frac{\partial}{\partial x_j}\left[\alpha_k \mu_{\text{eff}} \frac{\partial k}{\partial x_j}\right] + G_k + \rho\varepsilon \tag{9}$$

$$\frac{\partial(\rho \varepsilon)}{\partial t} + \frac{\partial(\rho \varepsilon u_i)}{\partial x_i} = \frac{\partial}{\partial x_j}\left[\alpha_\varepsilon \mu_{\text{eff}} \frac{\partial \varepsilon}{\partial x_j}\right] + \frac{C_{1\varepsilon}^{*}\varepsilon}{k}G_k - C_{2\varepsilon}\rho\frac{\varepsilon^2}{k} \tag{10}$$

Among them,

$$\mu_{\text{eff}} = \mu + \mu' \tag{11}$$

$$\mu_i = \rho C_\mu \frac{k^2}{\varepsilon} \tag{12}$$

$$C_\mu = 0.0845,\ \alpha_k = \alpha_\varepsilon = 1.39 \tag{13}$$

$$C_{1\varepsilon}^{*} = C_{1\varepsilon} - \frac{\eta(1-\eta/\eta_0)}{1+\beta\eta^3} \tag{14}$$

$$C_{1\varepsilon} = 1.42,\ C_{2\varepsilon} = 1.68 \tag{15}$$

$$\eta = \left(2E_{ij}\, E_{ij}\right)^{1/2}\frac{k}{\varepsilon} \tag{16}$$

$$E_{ij} = \frac{1}{2}\left(\frac{\partial u_i}{\partial x_j} + \frac{\partial u_j}{\partial x_i}\right) \tag{17}$$

$$\eta_0 = 4.377,\ \beta = 0.012 \tag{18}$$

Turbulence intensity $I$ is calculated by the following formula:

$$I = u'/\overline{u} = 0.16\left(Re_{D_H}\right)^{-1/8} \tag{19}$$

Among them, $u'$ and $\overline{u}$ are turbulent fluctuating velocity and average velocity respectively, and $Re_{D_H}$ is Reynolds number calculated by hydraulic diameter $D_H$. For a circular pipe, the hydraulic diameter $D_H$ is equal to the diameter of the pipe. For other geometric shapes, it is determined by the equivalent hydraulic diameter. Turbulence length scale $l$ is calculated by the following formula:

$$l = 0.07L \tag{20}$$

where, $L$ is the associated size. For fully developed turbulence, it is advisable that $L$ be equal to the hydraulic diameter. The turbulent viscosity ratio $\mu_t/\mu$ is proportional to the turbulent Reynolds number. Generally $1 < \mu_t/\mu < 10$ is preferable. The modified turbulent viscosity $\tilde{v}$ is calculated by the following formula:

$$\tilde{v} = \sqrt{\frac{3}{2}}\overline{u}Il \tag{21}$$

Turbulence kinetic energy $k$ is calculated by the following formula:

$$k = \frac{3}{2}\left(\overline{u}I\right)^2 \tag{22}$$

If the turbulence length scale $l$ is known, the turbulent dissipation rate $\varepsilon$ can be calculated by the following formula:

$$\varepsilon = C_{\mu}^{3/4} \frac{k^{3/2}}{l} \tag{23}$$

### 2.3 Computational grid and sensitive analysis

A tall building computed model is built as shown in Fig. 2. The windward and leeward region longitude is $5H$ = 400 m and $10H$=800 m, individually. The corresponded sizes of the region are $W \times D \times H$ =1200 m × 290 m × 400 m (Fig. 2) and the maximum blockage rate is 1.4% which is lower than the recommended largest blockage ratio (Tominaga et al. 2008b). The computational grid consists of almost 4,500,000 hexahedral cells. High quality mesh is imperative for the precise simulation of natural ventilation under ABL (Atmospheric Boundary Layer) wind flow condition. This study utilized the grid generation of grid extrusion operation method provided by Van Hooff and Blocken (2010). Figure 3 presents that the precise high-quality grids inside of the building and opening.

In this research, in order to balance the computation time and discretization error, grid sensitivity analysis is carried out. Figure 4 indicates the simulation results of three type grids which are coarse, middle and fine. Different grids are divided into three types based on a linear factor $\sqrt{2}$ . The coarse grids have 3.0 million and fine grids get 6.0 million grids, respectively. In addition, the minimum mesh size corresponding to the above three types ranges from 0.038 m, 0.027 m, and 0.014 m. Measurement lines locate at vertical and horizontal direction behind the building to compare different grids sensitivity. Figure 4 compares the wind speed distributions of the vertical lines at the gap and horizontal lines beside the pedestrian horizontal plane of the three grids. The results illustrate limited independence on the grid under the three types through WVA (wind velocity amplification factor) values. In this case, the mean difference between the medium and coarse grid along these lines is 0.8% while the fine grid is almost the same as the reference grid. Hence, the medium grid is chosen as the CFD simulated model.

The maximum and minimum grid volumes in the region are around 8 × $10^{-8}$ $m^3$ and 1.25 × $10^5$ $m^3$, respectively. The length from the center point of the wall adjacent cell to the wall, for the upstream, downstream, and ground are all about 0.020 m. In the voids of building, this length ranges from 0.02 m to 0.05 m and the corresponded $y^*$ values are from 30 to 300. These above values guarantee that the core of the grid near the wall is placed in the logarithmic layer so as to fully utilize the standard wall function.

### 2.4 Boundary configuration

The neutral air layer airflow profiles adopt the logarithmic law at the inlet opening of computational volume. The averaged wind velocity $U$ (m/s), turbulence dissipation ratio $\varepsilon$ ($m^2/s^3$) and turbulent kinetic energy $k$ ($m^2/s^2$) are implemented as follow equation (Richards and Hoxey 1993):

$$U(z) = \frac{u_{\mathrm{ABL}}^{*}}{\kappa} \ln\left(\frac{z+z_0}{z_0}\right) \tag{24}$$

$$k(z) = \frac{u_{\mathrm{ABL}}^{*2}}{\sqrt{C_u}} \tag{25}$$

$$\varepsilon(z) = \frac{u_{\mathrm{ABL}}^{*3}}{\kappa(z+z_0)} \tag{26}$$

where $z$ is the height, $\kappa$ is the von Karman constant (= 0.40–0.42), $u_{\mathrm{ABL}}^{*}$ is the ABL friction velocity and $C_u$ is a model constant of the turbulence model (Richards and Hoxey 1993). $z_0$ represents the aerodynamic roughness longitude and is estimated as a grass covered terrain which is 0.03 m (J. Wieringa). $u_{\mathrm{ABL}}^{*}$ is set as 3.4 m/s according to weather station data at the reference height of 10 m. The standard wall functions by Launder and Spalding (1974) with roughness modification are used (Blocken et al. 2007) with regard to the surface of ground. The physicals and particle roughness height $k_s$ (m) and the roughness constant $C_s$, are determined by the aerodynamic roughness longitude $z_0$ (Blocken et al. 2007). Such kind of relationship is:

$$k_s = \frac{9.793 z_0}{C_s} \tag{27}$$

At the surfaces of architecture, the standard wall functions are, also used but with zero harshness height $k_s$=0 ($C_s$=0.5).

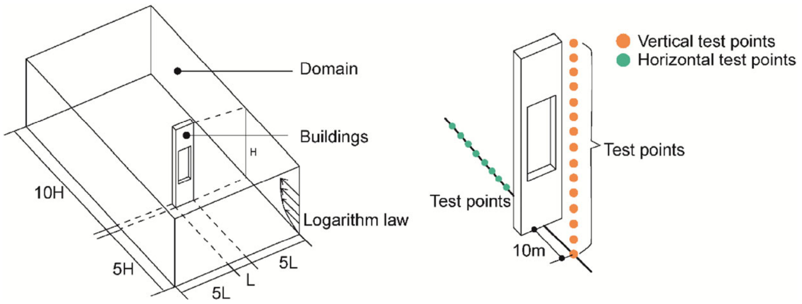

**Fig. 2** Computational domain sizes and test point locations

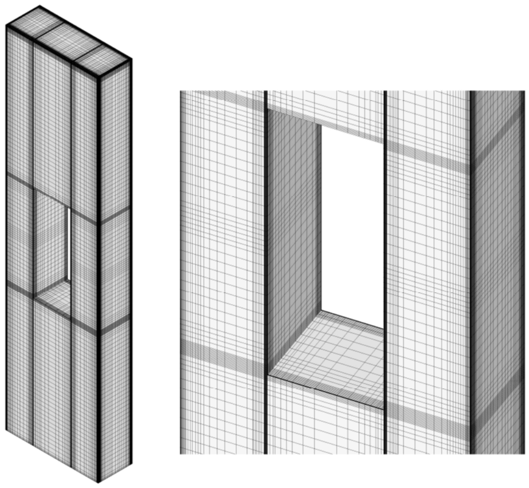

**Fig. 3** High resolution grid condition coupled with high-rise building generated by ICEM

The outlet boundary adopts zero gauge hydrostatic pressure and the other side of the whole area adopts the same structure.

### 2.5 Solver setting

The advanced turbulence model such as LES and DES could indeed predict wind flow field more accurately than $k$–$\varepsilon$ turbulence model for outdoor environment. However, the above two turbulence models require more accurate parameter settings such as sub-grid configuration and much more computational time than RANS CFD method. For LES, it has been proved to be superior than RANS model because of its high precision especially in terms of gustiness. For DES, it can also produce similar exact results with LES. Nevertheless, LES model needs high requirement for hardware and computational time (Shen et al. 2017; He and Song 1999). Moreover, DES requires higher mesh resolution and parameters (Liu et al. 2017a). In this study, the research condition cannot meet the requirement of the two above turbulence models. Therefore, this study implemented the RANS turbulence modelling method to perform the simulation. The RNG $k$–$\varepsilon$ model could improves the accuracy compared to standard $k$–$\varepsilon$ model and takes lower computed time and computer resources (Mittal et al. 2018). The final results also have a good agreement with the wind tunnel measurement consequences.

The 3D stable RANS operation equations and RNG $k$–$\varepsilon$ turbulence model were performed by isothermal CFD (Du et al. 2017) using of the business CFD code Fluent 17. This can be explained that its' general good performance for outdoor wind flow of buildings simulation. Pressure–velocity coupling employs the SIMPLE algorithm and pressure interpolation adopts second order format. As for convection current and sticky terms, second-order discretization schemes are put into them. Whole computational process would be terminated if the scaled residuals decrease to minimum values of $10^{-7}$ for $x$, $y$, $z$ momentum, $10^{-6}$ for $k$, $\varepsilon$ and continuity. Whole computations are executed on an 8-core station (Intel Xeon E5 2680 v3, 2.7 GHz) with 16 GB DDR of systematic memory.

## 3 CFD validation

This purpose of this research is to study the effect on wind flow condition of different auditorium patterns. Considering that the difficulties of field measurement for tall buildings' wind environment, the article utilizes the reference wind tunnel results for high-rise surrounding environment by scholar Tsang et al. (2012) to validate grid independence.

### 3.1 Boundary condition

A wind tunnel test was conducted by Tsang et al. (2012).

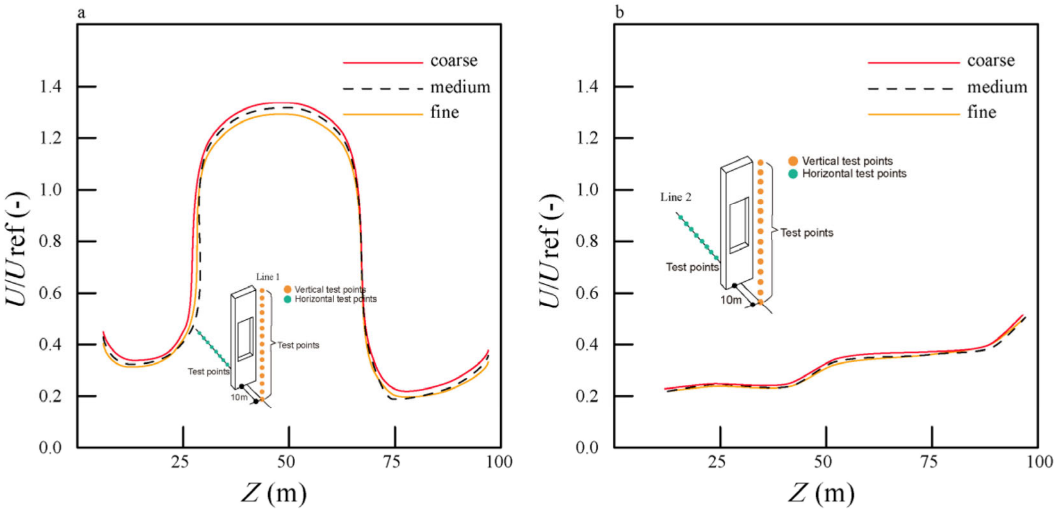

**Fig. 4** Grid sensitivity analysis of different resolution grids

As a case study, a 1:200 model test was conducted at CLP Power Wind Wave Tunnel Facility (WWTF) of Hong Kong University of Science and Technology. The wind tunnel is a device testing wind condition implementing for civil engineering etc. are coupled with a close circuit subsonic boundary layer (Fig. 5). It includes two measured parts: high and low speed section respectively. In present study, it was performed only in the high-speed testing section with a cross-section of 3 m × 2 m. Inlet averaged reference mean airflow velocity set as 10 m/s and the normalized wind speed WVA was used for validated the results between simulation and measurement. All building models were modelled under a scale of 1/200. In addition, as the wind tunnel just utilized for CFD validation, a single building model with fixed 25 m depth was only chosen for comparison with simulation result. Irwin sensors were used for perform indicators for whole wind tunnel measurement at height of 10 mm from ground equivalent to 2 m for building. They distributed around tested buildings at the upstream of 1.5*d*, and downstream of 15*d* (*d* means depth of building). Figure 6 shows the wind tunnel measurement and comparison of validation results (Tsang et al. 2012).

### 3.2 Validation results

Wind measurement equipment of wind tunnel and model positions are shown in Fig. 6(a). Figure 6(b) indicates the two test lines distribution in building surrounding in order to validate the simulation independence. Line A put at the downstream of building along the approaching wind direction. Line B is vertical with the inlet airflow direction at the behind of the building. Figure 6(c) shows the comparison of the simulated and measured mean wind amplified ratios $U/U_{ref.}$ It can be shown in Fig. 6(c) that both of points on two testing lines of CFD simulations show good agreement with wind tunnel measurement results especially in the area where is

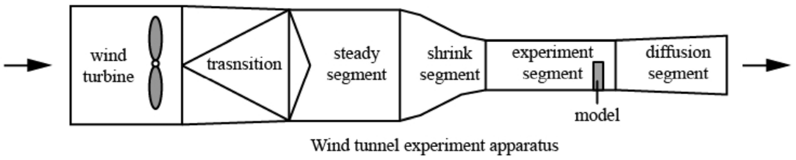

**Fig. 5** Wind tunnel experiment of Wind/Wave Tunnel Facility

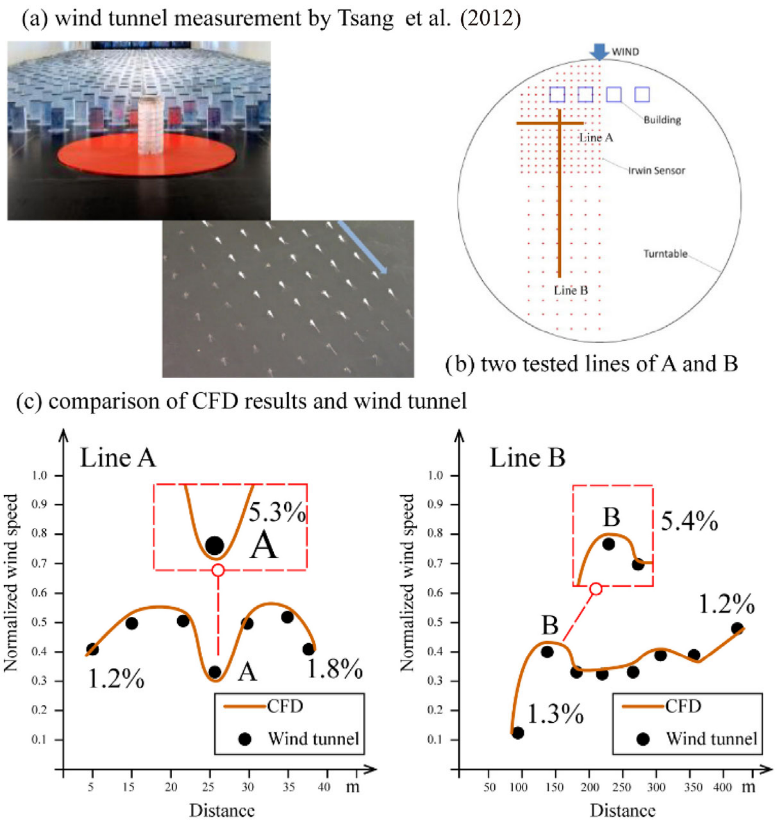

**Fig. 6** CFD validation with wind tunnel experiment: (a) wind tunnel measurement by Tsang et al. (2012); (b) two tested line schematic graph of A and B; (c) comparison of the results between simulation and wind tunnel measurement away from building. Although points locating behind near construction present larger errors than other points, the deviations of other sensors are all controlled within 5%. For example, the deviation value for points A and B is approximately 5.3%, 5.4% respectively, while that for other sensors mainly concentrates around 2%. Overall, this validation results are acceptable and this independent grid could use for following simulation.

### 4 Results and discussion

In this paper, 16 cases are simulated separately, and classified into two groups to focus on the following two respects:

- Under same holes area, the effect on the surrounding wind environment by different number of voids.
- Under same number of holes, the effect on the surrounding wind environment by voids distribution condition on building.

#### 4.1 The effect on the surrounding wind environment by different number of voids

It can be clearly seen from Fig. 7 that in order to investigate the impact on the surrounding building wind condition by

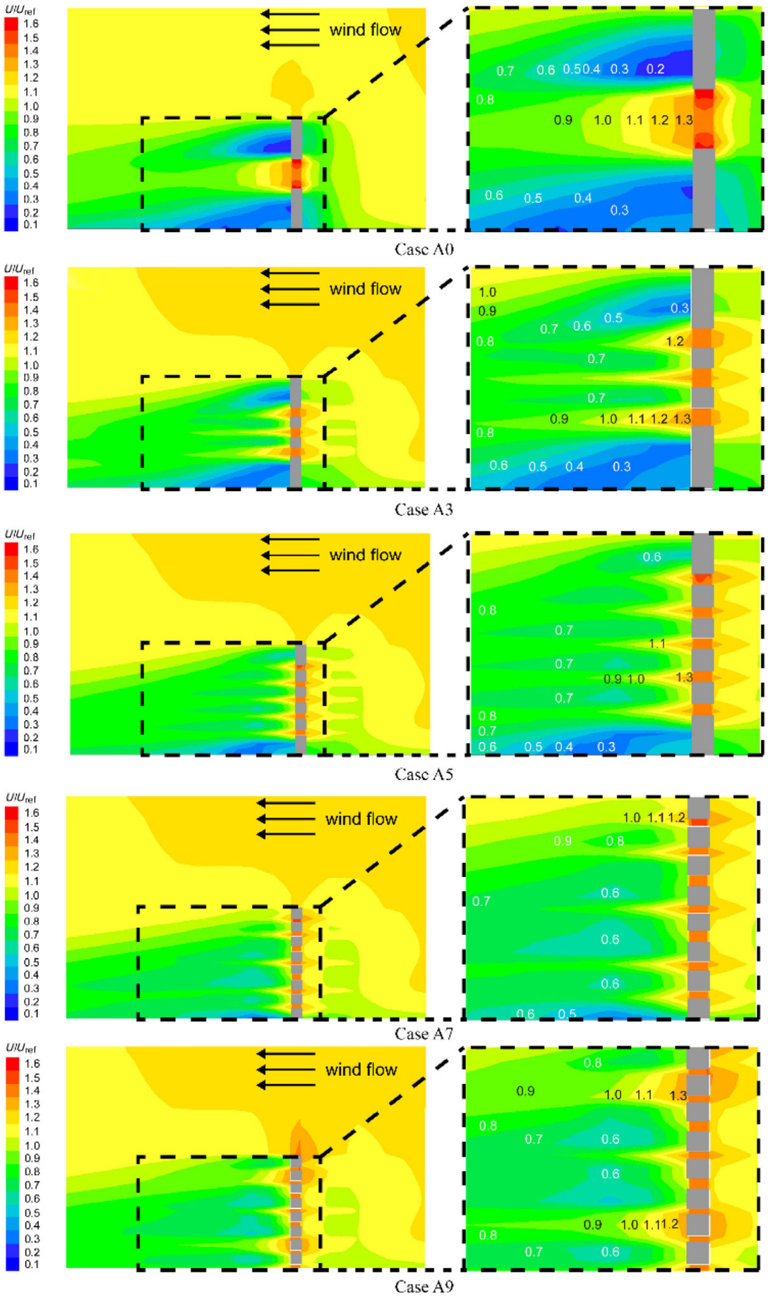

**Fig. 7** Wind flow field contour of case A with case A0, case A3, case A5, case A7 and case A9

voids numbers, the whole cavity in the building are split ranging from 3 to 9 under same amount hole area. The wind velocity amplification factor (WVA) regards as the assessment standard.

In general, along the height direction the wind speed becomes more evenly distributed as the holes number increases. Therefore, small and empty openings can effectively improve the wind velocity condition behind the high-rise building compared to a large whole cavity under same opening area. In this case, it is more conducive to disperse of pollutants and heat around the high-rise. To quantitatively assess the ventilation condition, the vertical lines are taken at the rear of the high-rise building center line 10 m, and the test points are picked up every 5 m along the vertical line to calculate the WVA values (Fig. 1).

##### 4.1.1 *Wind amplification effect along vertical direction behind building*

As can be seen from Figs. 8(a), (b) the WVA (wind velocity amplified factor) values of the case A are different with each other as the increased height. In case of a single void opening, the wind speed increases in the range of height within 40 m and it reduces at other heights. Under the condition of two holes and three holes, overall WVA enhances in the range of 20–80 m with larger than 1indicating that the building under this condition could be conducive to enhance the surrounding airflow quality. In addition, it can also be seen that the range of increase in wind speed is larger than that in a single void opening. Under the condition of four to five holes, the airflow velocity in the area behind the building is generally enhanced within the entire height range, and the wind speed amplification factor fluctuates around 1. In these cases, the vertical wind environment is the best, which is beneficial to the improvement of the airflow environment after the wind blows through the high-rise buildings. Therefore, compared with the one opening, the case of multi-voids can effectively increase the wind speed in the wind shadow area, promote the loss of pollutants and heat, which is conducive to the solution of the heat island effect problem.

##### 4.1.2 *Wind amplification effect along horizontal direction behind building*

In order to further investigate the influence of the wind on the surrounding wind height environment under different conditions of the number of openings. To set different points along a horizontal line at the height of 1.5 m to record the wind speed locating at the centre of each model. As can be seen from the analysis of Figs. 8(c), (d) at the pedestrian height the increase of the number of voids is beneficial to the amplification of the wind speed at the pedestrian height and the improvement of the wind environment. For example, the wind amplification factor of the case A9 hole is the best, and case A0 is the worst. Moreover, under the condition of 7, 9 concavities, the maximum value of the WVA is also the biggest among all models. For case A0, the wind amplification coefficient of the pedestrian height at the back of the building

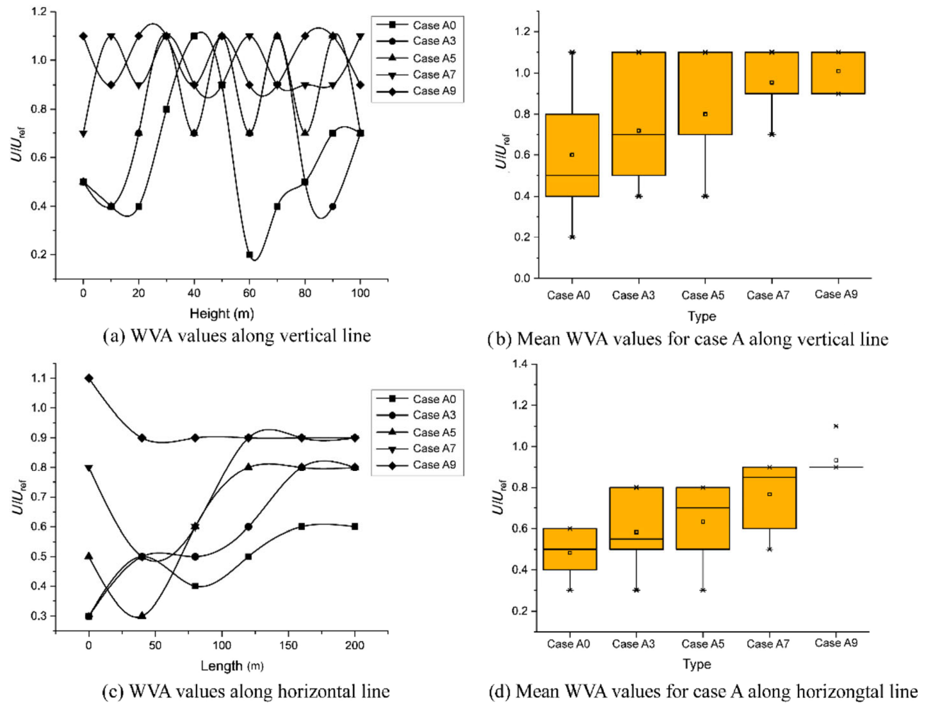

**Fig. 8** WVA (wind velocity amplified factor) statistics of the case A: (a) WVA values along vertical line; (b) mean WVA values for case A along vertical line; (c) WVA values along horizontal line; (d) mean WVA values for case A along horizongtal line is small, which is less than 1, indicating that the wind speed diminishes apparently after flow blowing through the building. Furthermore, for cases A7 and A9, WVA values starts to increase around 75 m, and reaches the extreme value when it is about 125 m. Although the extreme value is still less than 1, it is far more than cases A0 and A3.

It can be seen from the above analysis that the installation of the opening in the high-rise building can effectively improve the wind environment after airflow blows through the building. In the case of same opening area, the more the number of holes, the more the wind velocity in the vertical direction of the building can be strengthened, and the wind environment in the vertical direction distributes more stable and evenly. Simultaneously, the increase in the number of holes can effectively improve the length of the wind shadow area in the rear of the building, reduce the length of the wind shadow area, and weaken the degree of wind speed reduction.

### 4.2 The effect on the surrounding wind environment by voids distribution

As shown in Fig. 9, in the case where the total area of the void is the same, the hole is divided into three equal parts, and they are symmetrically distributed along the building.

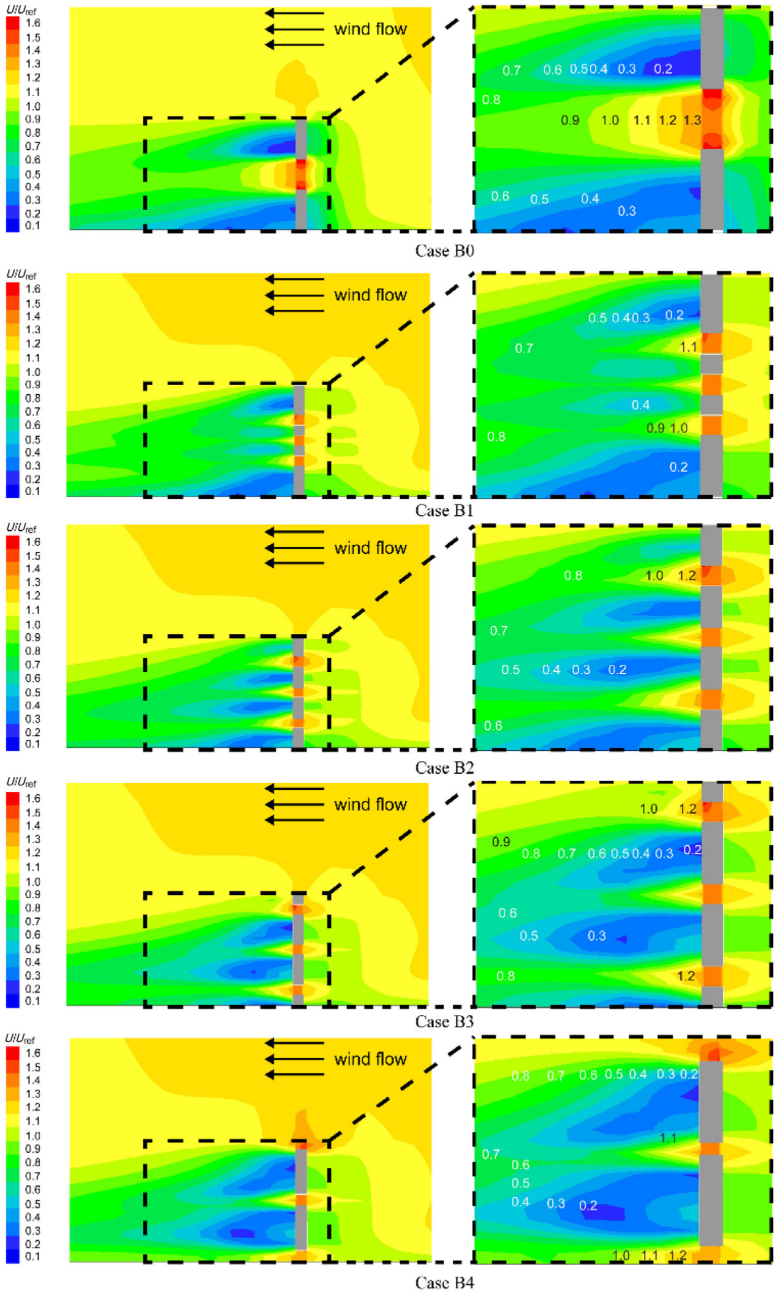

**Fig. 9** Wind flow field contour of case B with case B0, case B1, case B2, case B3 and case B4

In general, with the increase of $R$ which is defined the ratio of the gap between two voids to the height of holes, the WVA in the wind shadow area at the rear of the building tends to increase first and then decrease. In the vertical direction, as $R$ increases, the wind speed distribution presents a phenomenon of average first and fluctuation then. The length of the wind shadow zone along the horizontal direction also exhibits an unsteady situation with the change of $R$.

#### 4.2.1 *Wind amplification effect along vertical direction behind building for case B*

As can be seen from Figs. 10(a), (b), the wind speed distribution along the height direction fluctuates greatly. It can be seen that as the distribution of the openings along the vertical direction of the building is more discrete, the distribution of wind speed along the height direction in the wind shadow area behind the building will vary greatly. When $R$ ranges between 1 and 3, the more uniform the wind speed distribution in the vertical direction of the building wind shadow area, the more stable the wind environment, and the smaller the wind loss. When $R$ is less than 1 and greater than 3, the wind speed distribution in the wind shadow area is more unstable, and a larger wind shadow area will occur. The airflow velocity amplification factor will also be somewhat smaller, which is not conducive to heat loss. For example, When $R$ is 0, it can be clearly seen that the wind speed amplification factor is gradually increasing in the range of 30–60 m. This is because the three openings are concentrated in the middle of the building, so the wind amplification effect at this place is the best. When $R$=4, the increase of the WVA values mainly concentrate in the range of 10–20 m, 40–50 m, and 90–100 m which distributes more discrete.

#### 4.2.2 *Wind amplification effect along horizontal direction behind building for case B*

In the horizontal direction, the distribution of the hole becomes more discrete, the wind shadow area at the pedestrian height changes to be smaller and the wind speed amplification factor gradually increases (Figs. 10(c), (d)). For example, at 1.5 m pedestrian level, when $R$ is 1, 2, the wind speed amplification factor distribution is similar in both cases, and the wind speed amplification factor reaches the maximum after 100 m. Compared with the case where $R$ is 0, it can be seen that when $R$ is 0, the wind speed amplification factor reaches the maximum after 150 m. The wind environment reaches a maximum within a short distance, which is beneficial to improve the wind environment at the walking height.

In addition, since this simulation only considers the case of the holes locating at the middle position, so the next simulation analysis is performed on the case where the hole is located at both ends.

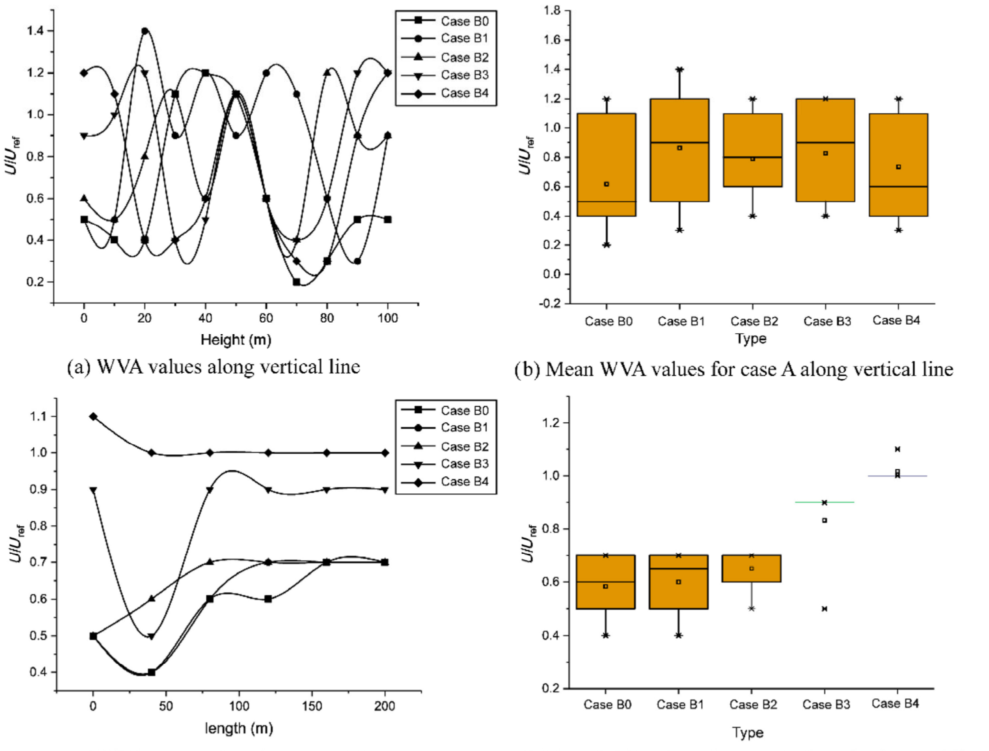

(a) WVA values along vertical line (b) Mean WVA values for case A along vertical line

(c) WVA values along horizontal line (d) Mean WVA values for case A along horizongtal line

**Fig. 10** WVA (wind velocity amplified factor) statistics of the case B: (a) WVA values along vertical line; (b) mean WVA values for case A along vertical line; (c) WVA values along horizontal line; (d) mean WVA values for case A along horizontal line

#### 4.3 The effect on the surrounding wind environment by end of the voids

It can be seen from Fig. 11 that in the case where the number of the opening areas is the same, when the lower end opens the hole, the surrounding wind environment will change correspondingly with the difference in the position distribution of the opening. In general, when the lower end opens the hole, the wind environment at the bottom is the best, the wind speed amplification factor is the largest, and the position where the air is not permeable will form the corresponding wind shadow area. As the hole is distributed upward, the hole is gradually dispersed, and the number of wind shadow areas increased, but the area is decreased.

##### *4.3.1 Wind amplification effect for case CU*

As can be seen from Figs. 11, 12, when the upper end is open, the lower end is not permeable to the wind, and the wind shadow area is the largest. The wind speed amplification factor at the upper end is the largest. As the hole moves downward, the range of the lower wind shadow area gradually decreases, and the range along the horizontal direction also gradually decreases, but the upper part gradually produces a wind shadow area along a vertical direction. The distribution of the upper wind speed gradually tends to fluctuate.

##### *4.3.2 Wind amplification effect for case CD*

As can be seen from the analysis of Fig. 12, when the RD is 0, the wind speed at 40 m is well amplified, and the wind speed amplification factor tends to 1. After 40 m, the overall wind speed is blocked, so the wind speed amplification factor is very low. When RD is 1, the WVA at 60 m is similar to that when RD is 0, but at this time, the fluctuation range of the wind speed amplification factor is increased. When RD

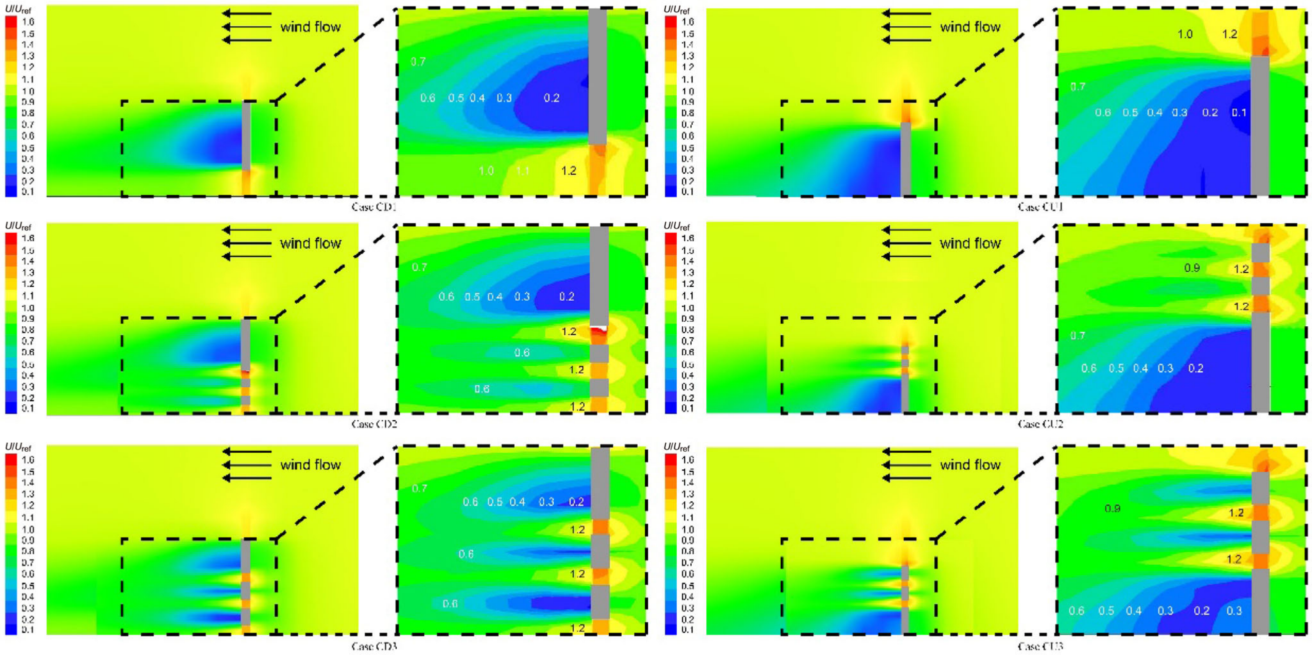

**Fig. 11** Wind flow field contour of case C with case CD and case CU

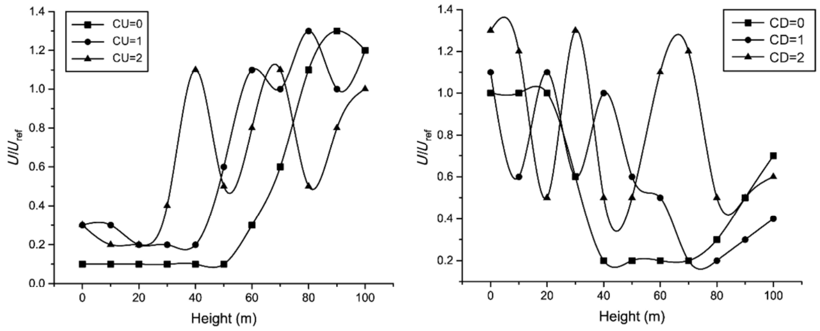

(a) WVA values along vertical line of case CU (b) WVA values along vertical line of case CD

**Fig. 12** WVA (wind velocity amplified factor) statistics of the case C: (a) WVA values along vertical line; (b) WVA values along horizontal line is 2, the distribution of the opening is more dispersed, and the fluctuation range of the wind speed amplification factor is further increased. The wind speed within the height of 80 m is fluctuating. As the number of RD enhances, the numbers of wind shadow areas further increase. Nevertheless, even if the wind shadow area is increased, the wind speed amplification factor of the upper wind shadow area is far larger than the far end.

##### 4.3.3 *Comparison of wind amplification effect for case CD and case CU*

Figure 13 shows that in the case where the upper end is open, the change in the wind speed is almost opposite to the lower end opening. When the RU is 1, the average value of the wind speed amplification factor is the largest, and the fluctuation amplitude is relatively small, because that the hole dispersion degree in this case is moderate, and the influence range is large with a larger wind shadow area. When the lower end is open, it can be seen that the mean wind speed amplification factor is the same when RD is 1 and 2. But for case RD1, the overall distribution is more uniform and the fluctuation amplitude is smaller. Therefore, when RD is 1, for the lower end opening, the wind environment is the best, which is conducive to building energy conservation and heat loss.

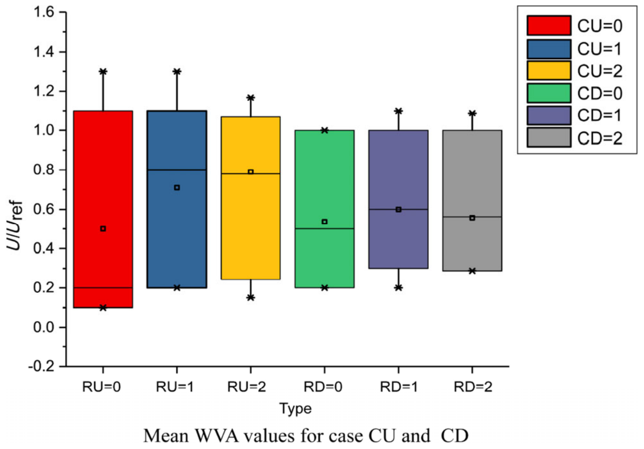

**Fig. 13** Comparison of mean wind speed amplification factors between upper end and lower end openings

#### 4.4 Effects on air pollutant dispersion by holes on the high-rise building

Figure 14 presents the air pollutant distribution condition for cases A, B and C. In order to quantitatively assess the contaminant flow field situation, a concept of pollutant concentration ratio (CR) is defined as follows:

$$\mathrm{CR}=\frac{\text{pollutant concentration of inlet wind (approch airflow)}}{\text{pollutant concentration along the vertical measurement line}}$$

A vertical measurement line is positioned 10 meters in front of the building to investigate the pollutant concentration. Along this vertical direction, $z$ represents the height of testing point and $H$ indicates the building height (Fig. 14). When CR value is larger than one, the better the pollutant dispersion situation (Figs. 14, 15). Following observations can be made:

- With the number of voids increase, the air contaminants disperse more efficiently. The best pollutant dissemination condition is achieved when the holes almost completely distribute along the building i.e. case 9A. This is mainly because that the air pollutants locating at the leeward of building can be flowed by the wind through the hollow areas. Consequently, more construction voids can promote efficiently the loss of pollutants situating downstream of the building. For example, in cases of A7 and A9, all CR values are under 0.5 which is apparently smaller than the averaged CR value of 2.38, 2.11 and 1.27 for cases A0, A3 and A5, respectively.
- The distribution of air contaminants is related to the building voids positions. The distance between hollow areas on the building could significantly influence the pollutant gathering situation in the leeward of construction. For distance between holes on building larger than twice height of holes such as case B3, the pollutant gathering effect at the behind of gap between voids enhances increasingly by enlarging the distance between holes. On the other hand, it should be noted that for cases B0 to B2, air contaminants behind the gap between holes could also be dispersed effectively. For example, CR values of 0.7 and 1.8 for case B2 at the height of $z/H$ being 0.3 and 0.7 are much lower than that under same height for cases B3 and B4 which are 4, 5, 4, 5 respectively.
- For wind flow condition of hollow volumes where position at the end of building (case C), the discretization of cavities in building could apparently improve pollutant dispersion condition behind architecture wherever the voids locating at the top or bottom of construction. For example, with regard to case C, the $z/H$ range under 1.5 of CR value for case CU1 locates at 0.7, which is significantly larger than that of 0.5 for case CU3. This result distinctly shows that the pollutant condition is improved around the middle of building by increasing the opening discrete degree. It is an important conclusion, especially in view of sky court which increasingly occurs in the roof of high-rise building, the decomposed distribution for this air garden could effectively enhance the status of loss of pollutant dispersion in the middle of high-rise building.

### 5 Discussion

For wind energy harvesting, many relevant researches have

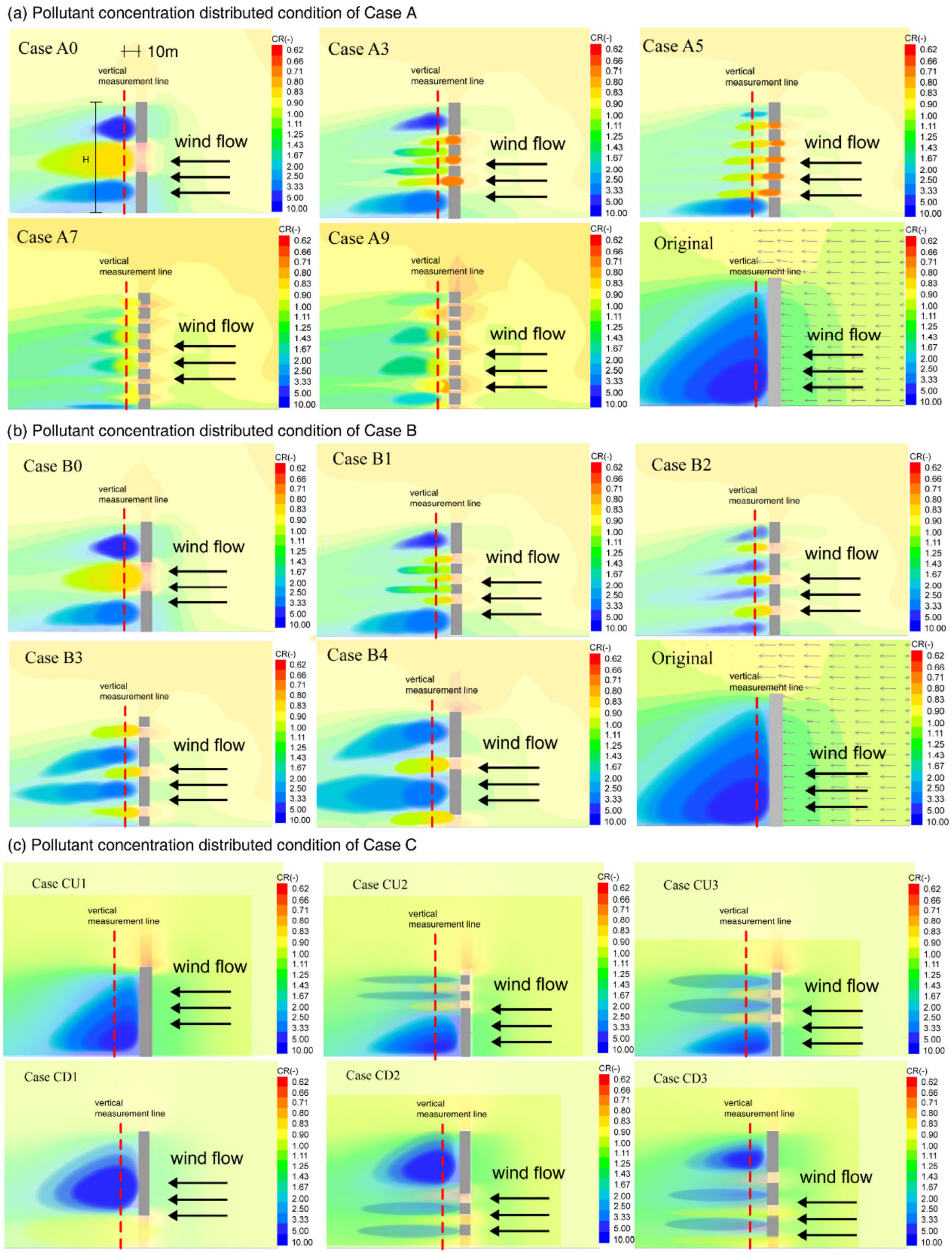

**Fig. 14** Pollutant concentration contour for three cases: (a) pollutant concentration distributed condition of case A; (b) pollutant concentration distributed condition of case B; (c) pollutant concentration distributed condition of case C

been performed to investigate the potential of opening using in high-rise building. Ayhan and Sağlam (2012) found that implanting cross-holes in building is an effective approach to enhance wind velocity to utilize wind energy. In addition, voids in building also can be considered as refuge storey which is indispensable for high-rise building. Hence, this dual purpose of openings has increasingly received attention from scholars due to its economic and environmental friendly.

For wind energy utilization, most studies mainly focused

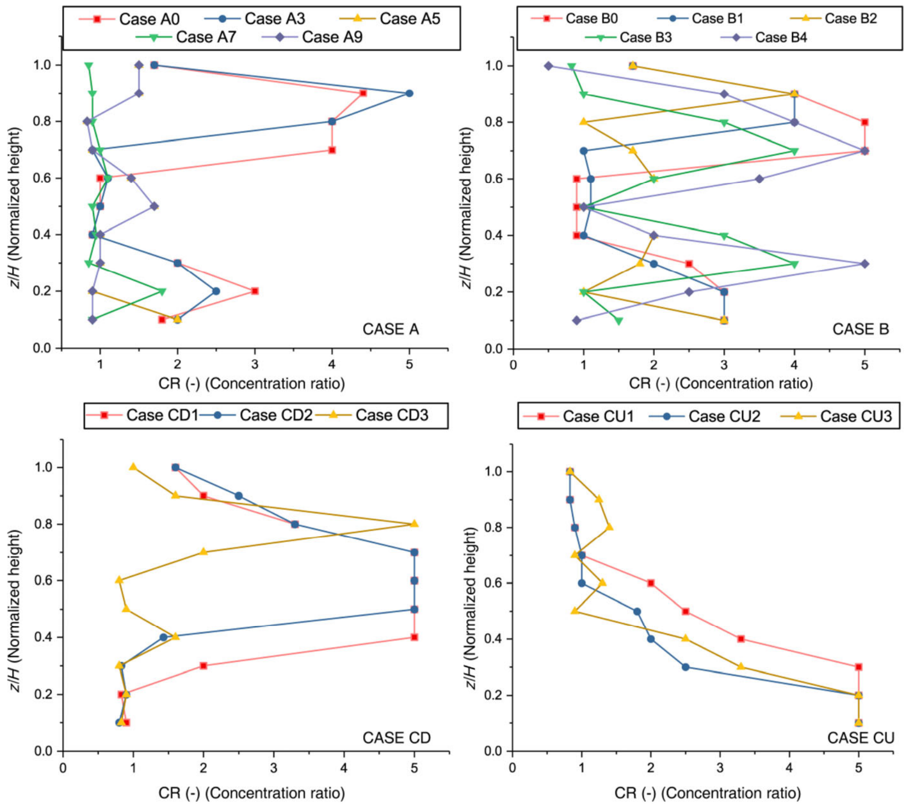

**Fig. 15** Concentration ratio of pollutant statistics along building height for cases A, B and CU, CD

on the pattern of building hollows integrated with wind turbines. Li et al. (2013) investigated the Pearl River Tower wind energy harvesting condition using wind tunnels and indicated that building orientation, incident wind angle and inlet opening shapes have significant influence on the airflow velocity via hollows. In addition, Li et al. (2016) also found that upper corridor could better amplify the airflow velocity for wind turbines. Hassanli et al. (2017) designed a special cascade wind turbine which can utilize wind energy in high efficiency mounted inside the cross opening in high-rise building. Hassanli et al. (2019) investigated the effect of different cross holes configurations on the wind flow characteristics influencing wind energy harvesting (Fig. 16(a)). It concluded that the mean airflow velocity in holes can be enhanced by 25% if the recessed area is set at the inlet and outlet of opening and the curved wall is arranged at the corners. Apart from this, as the convergence - divergence channel is implemented, the average velocity increases by about 33%. Zhou et al. (2017) also performed some similar researches in terms of the opening patterns (Fig. 16(b)). The results showed that the composite prism diffuser pattern of holes could apparently amplified the wind velocity and the best aspect ratio for wind harvesting using this shape is 0.6.

In addition to the above academic research, method of openings integrated wind turbine has been applied to practical projects. Figures 16(c), (d), (e) present the case of Bahrain World Trade Centre, Castle House in London and Pearl River Tower. As the first wind energy integrated building all over the world, three 225 kW horizontal-axis wind turbines are mounted on the connecting bridge of Bahrain World Trade Centre in the tunnel shaped between two skyscrapers. The two particular shapes of buildings apparently lead to the large accumulated wind effect. In case of Castle House skyscraper in London, three horizontal axis wind turbines with 9 m diameter are used at the top of this building to meet the requirement of the entire mansion lighting. For Pearl River Tower in China, the voids are constructed for double goals of wind energy harvesting and refuge space. There are four tunnels shaped on the building with bell-mouth pattern at the inlet and outlet of openings as shown in Fig. 16(c). Simultaneously, the concave wall on the south side and the convex wall on the north side could effectively magnify the wind speed and promote air to penetrate the tunnel. In this case, helix-shape vertical axis wind turbines mounted in every hollow can take fully advantage of wind energy to support whole mansion.

In accordance with above analysis, research done so far generating wind energy from hollows in building mainly focuses on the configuration of openings. However, the section shape and height of openings, the incident wind angles also have crucial impact on the wind flow characteristics. Therefore, further research should be performed to investigate

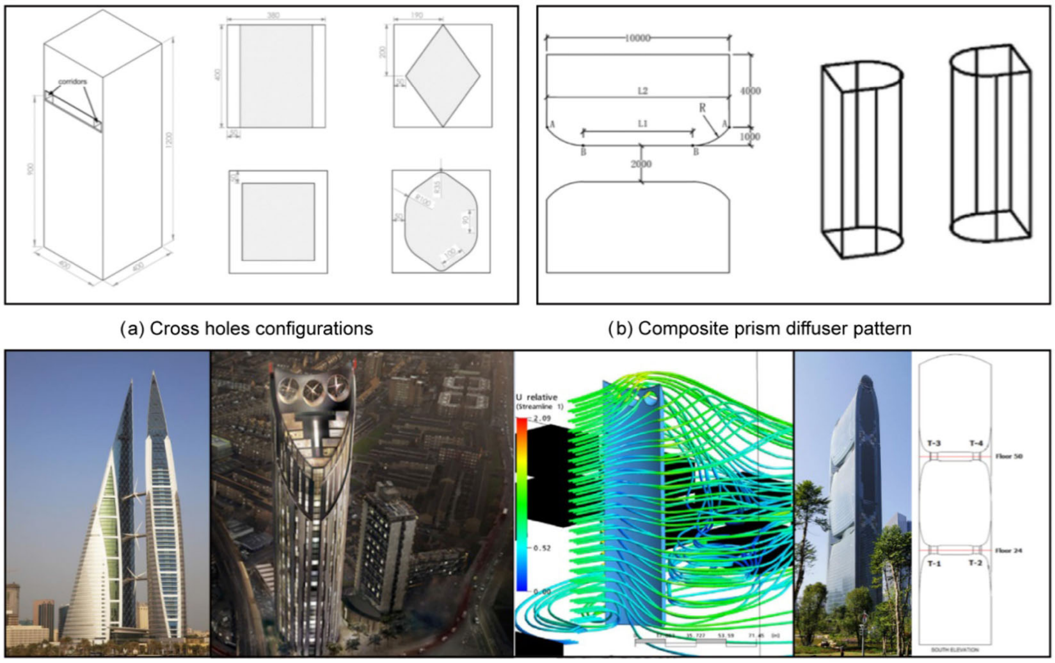

(a) Cross holes configurations (b) Composite prism diffuser pattern

(c) Bahrain World Trade Centre (d) Castle House in London Tower (e) Pearl River Tower

**Fig. 16** Pollutant concentration ratio statistics of different cases the relationship between flow field performance and opening section pattern, approaching wind directions.

On the other hand, for present study, porous model is another feasible method for performing this research. Hirano et al. first proposed using porous model to investigate building thermal characteristic in view of decreasing computational resources. In this research, holes in buildings were utilized to enhance the natural ventilation rate so as to diminish the cold load consumption (Hirano et al. 2006a,b). The void's ratio was estimated as the percentage of the holes of building scale in the whole building volume. Yan Liu et al. conducted a porous model to simulate the night ventilation feature of building and indicated that outdoor temperature had much more impacts on the night ventilation than the airflow speed (Liu et al. 2017b).

After that, taking consideration of large scale urban circumstance, porous model started to be used for performing natural airflow characteristic under city scale. Hang and Li (2010, 2012) regarded whole urban area as a combination of porous and fluid model to investigate city thermal and airflow environment. In addition, a new code called "Ventair" code based on FORTRAN language was created to check this porous model. On this scale, Wang et al. (2017) also adopted a mixed model of fully resolved and porous model to simulate the urban thermal and wind environment. All buildings and surroundings were set as porous model. Apart from this, so as to simplified simulation process, Antohe and Lage (1997) established a two-equation porous model which is identical with standard $k$–$\varepsilon$ model in terms of the closure coefficients.

In line with the above analysis, research done so far about porous model in building simulation mainly focused on large scale level such as urban planning. It is insufficient for investigations on single construction scale such as single building. Compared with the solid model, the porous model takes up less computer resources and lower grid resolution which significantly influences the computational time. However, the indispensable complex configuration of porous model shows difficulties for non-professionals such as building designers as well. Hence, future researches should be performed on application of porous model on single building thermal and ventilation characteristic and simplification of porous model simulation.

### 6 Conclusion

After research and analysis, it can be seen that the interior of the high-rise building can effectively improve the surrounding wind environment by opening holes. The same number of hollow volumes can also effectively affect the surrounding wind speed by adjusting the distance between one void to the others, thus affecting the heat loss around the building and the loss of pollutants. Not only that, but through the combination of modular high-rise buildings, a variety of hollow forms can be easily formed, and a variety of sky gardens can be formed to provide a more comfortable living environment.

Specifically, the following conclusions can be drawn:

1) Setting the void spaces in the high-rise building can effectively improve the wind environment after the wind blows through the building. In the case of the same holes area, the more the number of voids, the more the wind speed in the vertical direction of the building can be strengthened, and the wind environment in the vertical direction can be more stable and the overall distribution is more even.

2) The increase of the number of holes can effectively improve the length range of the wind area behind the building effectively improving the wind environment at the pedestrian height and pollutants lost.
3) When the ratio of the gap between the openings and its height ranges between 1-3, the wind environment in the vertical direction is the most stable, and the wind velocity attenuation is the smallest, which is beneficial to the loss of heat.
4) In the horizontal direction, as gap between the holes and the height of the voids increases, the distribution of the hole becomes more discrete, the wind shadow area at the pedestrian level becomes smaller, and the wind speed amplification factor gradually increases.
5) As the hole is arranged at the upper and lower ends, the wind environment at the pedestrian height will be better and better, and the wind speed amplification factor increase apparently, which is conducive to the loss of heat and pollutants.
6) Whether the building is open at the upper end or the lower end, as the hollow volume is gradually distributed downward, the wind environment in the horizontal direction will gradually improve as the distribution of the voids increase. Therefore, it should be fully considered when designing the building.

### Acknowledgements

This research has received funding from the European Union's Horizon 2020 research and innovation program under grant agreement No. 768735.

**Open Access:** This article is licensed under a Creative Commons Attribution 4.0 International License, which permits use, sharing, adaptation, distribution and reproduction in any medium or format, as long as you give appropriate credit to the original author(s) and the source, provide a link to the Creative Commons licence, and indicate if changes were made.

The images or other third party material in this article are included in the article's Creative Commons licence, unless indicated otherwise in a credit line to the material. If material is not included in the article's Creative Commons licence and your intended use is not permitted by statutory regulation or exceeds the permitted use, you will need to obtain permission directly from the copyright holder.

To view a copy of this licence, visit http://creativecommons.org/licenses/by/4.0/.
